# The Work of the Holy Ghost

**Published:** 1887-07-09

**Authors:** 


### Words of Consolation.

[Communications for this column should be addressed, "Chaplain," care of the Editor of The Hospital, who will gratefully welcome any suggestions or inquiries from matrons, nurses, and the staff generally.]

XL.—The Work of the Holy Ghost.

We have seen that just in proportion to the restoration of the Divine image in the soul, so does conscience become subject to the guidance of the Spirit, and regain its lofty position. The compass of life is repaired, so the needle points continually in the right direction.

Now let us mark this. It is when conscience is thus subject to the Holy Ghost that the real value of prayer is discovered. "He will guide us into all truth." But we must for ever be asking Him to direct and rule our hearts. Whenever we are in doubt, or difficulty, or perplexity, we must ask at once for the Spirit of Counsel, nothing doubting, and then simply follow, in child-like faith, whatever He suggests. We must, however, leave Him to choose His own way of directing us. This may be sometimes through a passage of Scripture, or by a single verse, which He may bring home to us with overwhelming persuasion. Sometimes, instead of giving us direct counsel, He may urge us to seek advice from one of God's ministers, or from a trusted friend. Sometimes the much needed counsel will come through suggestions made immediately to the soul. He reveals Himself in answer to earnest prayer; and the thoughts supplied, if acted upon, all tend to the making up of what is called a good conscience. If you are going along a narrow, dangerous path on a dark night, with a number of turnings and slippery places here and there, and you have taken a guide well acquainted with every one of these hindrances, you will not fail to appeal to him whenever you feel the slightest doubt about your next step as you move on. So is the *habit* of the soul guided by the Holy Ghost. Faith knows of no waiting to ask "which way," but asks at once, though she may sometimes wait until she is certain of having received direction. How well the prayer of faith in the Holy Ghost is both expressed and answered in the following words:—"Be thou also my guide, and lead me for thy name's sake" (Ps. xxxi, 3). "I will inform thee and teach thee in the way wherein thou shalt go, and I will guide thee with mine eye" (Ps. xxxii, 9). "Thou shalt guide me with thy counsel, and after that receive me into glory" (Ps. lxxiii, 24). "He shall be our guide unto death" (Ps. xlviii, 13). What a prophecy that is of Isaiah's, of conscience once darkened becoming enlightened by the spirit! "Say to the prisoners, Go forth; and to them that are in darkness, Show yourselves. . . . They shall not hunger nor thirst, neither shall the heat nor sun smite them; for he that hath mercy on them shall lead them, even by the springs of water shall he guide them" (Isaiah xlix, 9, 10).

Here, too, is a promise especially framed to meet the cases of some for whom we write:—"If thou satisfy the afflicted soul: then shall thy light rise in obscurity, and thy darkness be as the noon day: and the Lord shall guide thee continually" (Isaiah lviii, 10, 11).

(*To be continued.*)

